# No increased rate of cyclops lesions and extension deficits after remnant-preserving ACL reconstruction using the sparing technique

**DOI:** 10.1186/s13018-022-03356-2

**Published:** 2022-10-21

**Authors:** Sebastian Bierke, Martin Häner, Katrin Karpinski, Tilman Hees, Wolf Petersen

**Affiliations:** Department of Orthopedic and Trauma Surgery, Martin Luther Hospital, Caspar Theys Strasse 27-31, 14 193 Berlin, Germany

**Keywords:** ACLR, Remnant augmentation, Cyclops lesion, Arthrofibrosis, Outcome

## Abstract

**Background:**

Remnant-preserving anterior cruciate ligament reconstruction (ACLR) should have advantages for postoperative remodeling and proprioception. However, it has been suggested that the larger diameter of the graft tends to lead to impingement phenomena with a higher rate of cyclops lesions. The aim of this work was to find out whether the remnant-preserving ACLR actually leads to an increased rate of range of motion restraints compared to the remnant-sacrificing technique.

**Methods:**

Patients, who fulfilled the inclusion criteria, were followed up for one year after surgery. The primary endpoint was arthrolysis due to extension deficit or cyclops syndrome.

Secondary outcome measures were pain (NRS), knee function (KOOS), patient satisfaction and return to sports rate.

**Results:**

One hundred and sixty-four patients were included in the study, 60 of whom received the “remnant augmentation” procedure (group 1). In the remnant augmentation group, one cyclops resection was performed, whereas in the non-remnant augmentation group three cyclops lesion resections had to be performed (odds ratio 0.6). There was no difference between the groups in pain (NRS) and knee function (KOOS) and patient satisfaction. The return to sports rate after one year was higher in the remnant augmentation group.

**Conclusions:**

Patients who have undergone the sparing “remnant augmentation” ACLR have no increased risk of cyclops lesion formation or extension deficit in the first year after surgery. An improvement of the proprioceptive abilities by remnant augmentation ACLR should be investigated in further studies.

**Level of evidence:**

III (prospective cohort study).

## Introduction

The cruciate ligament rupture is a serious injury to the knee joint, which can lead to secondary meniscus and cartilage damage. In the long term, untreated anterior instability (giving way) can lead to osteoarthritis [19].

**Fig. 1:**
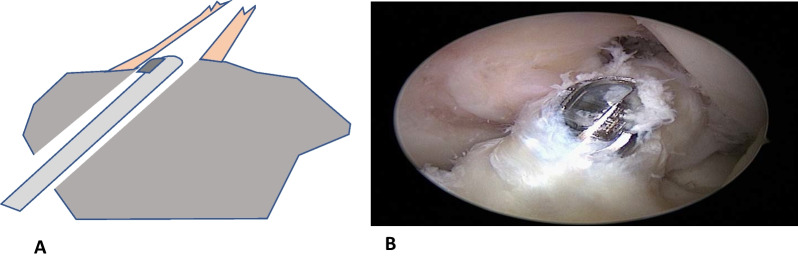
Surgical technique of the sparing technique for remnant-preserving ACL reconstruction. **A** Schematic drawing showing how the volume of the tibial insertion is reduced with the shaver in the tibial tunnel, **B** arthroscopic aspect

Therefore, the therapeutic goal should be to stabilize the knee joint again to prevent giving ways [25]. In patients who cannot compensate for the missing passive stabilizer neuromuscularly (non-coper), the replacement of the anterior cruciate ligament with an autologous or allogeneic tendon graft has been considered the gold standard [25]. In conventional ACL reconstruction, the insufficient ligament is debrided, removed and replaced by the tendon graft which is fixated in bone tunnels in the anatomic insertions at the femur and tibia [25]. Advantage of the so-called remnant-sacrificing technique is that the surgeon has a good overview of the femoral insertion zone (Fig. 1).

**Fig. 2:**
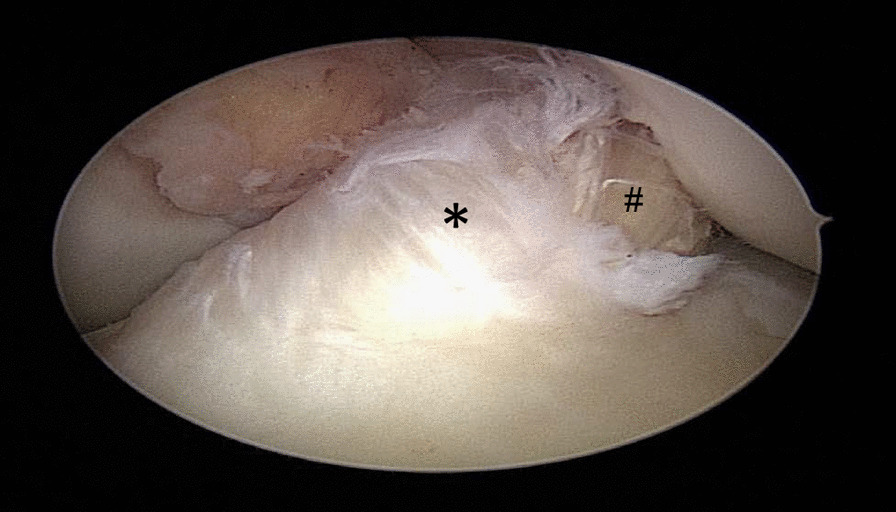
After finishing the remnant-preserving ACL reconstruction, the tendon graft is completely covered by old ACL tissue. Asterisk: ACL remnant, Rhombus: tendon graft

In recent years, however, the interest in biological solutions for the treatment of anterior ACL ruptures has grown. In addition to refixation or dynamic intraligamentary stabilization [1, 2], these biological solutions also include remnant ACL reconstruction [10, 18, 23, 29]. A schematic representation of the procedure is shown in Figs. 1 and 2.

**Fig. 3:**
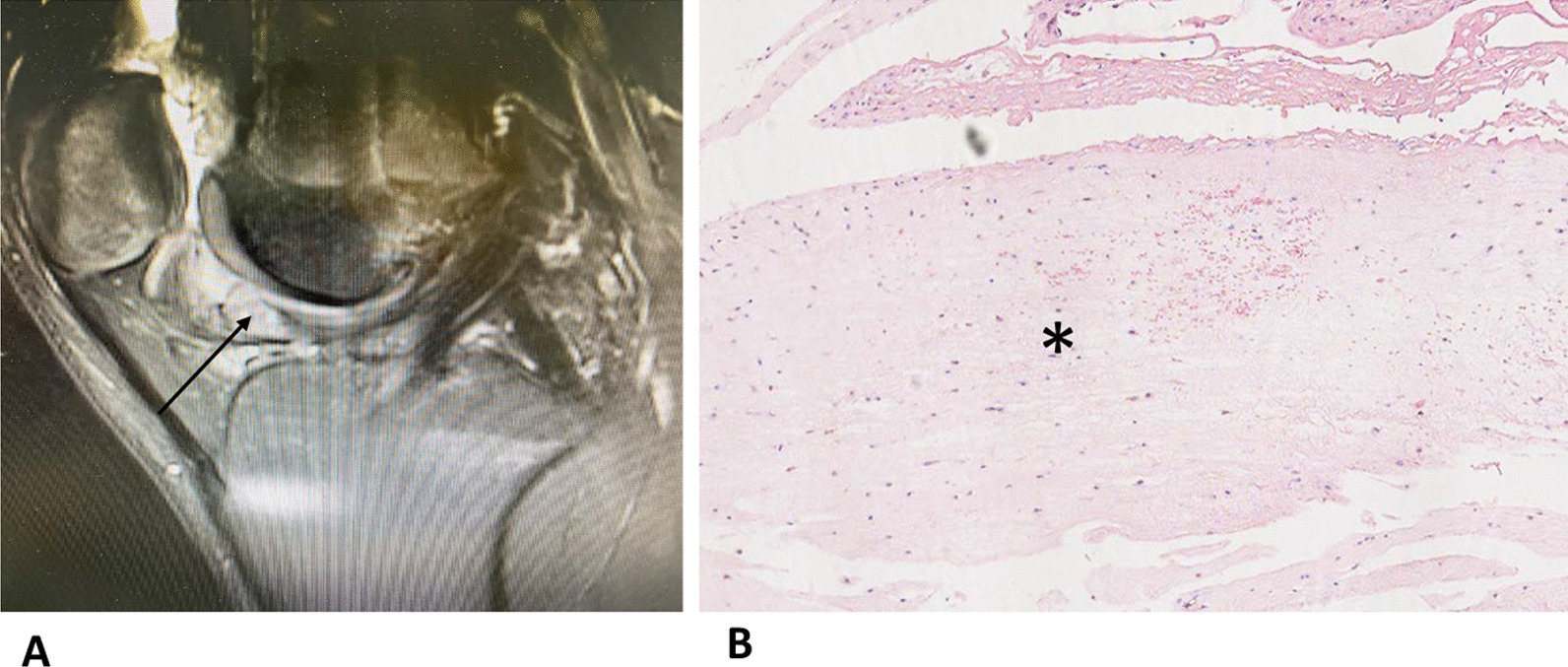
**A** MRI showing a cyclops lesion after ACL reconstruction (arrow); **B** dense connective tissue resembling tendinous tissue was detectable in all cyclops lesions (asterisk)

In remnant-preserving ACLR as much original ligament material as possible is left in the joint. The rationale for this technique is that a histological study found competent structures with well vascularized connective tissue, numerous fibroblasts and mechanoreceptors in the remnant tissue [15, 23]. Second look arthroscopies at one year postoperatively have shown that preservation of the ACL remnant with good synovial coverage had a positive effect on graft synovialization and graft integrity [15]. Current meta-analyses have shown that the remnant reconstruction technique leads to higher stability and better functional results [30]. The higher stability can probably be attributed to a larger diameter of the graft remnant construct as shown in a study by MRI [3]. A possible downside of the larger graft diameter could theoretically be a higher rate of impingement incidents of the graft to the roof of the femoral notch and an increased incidence of cyclops-like mass lesions [18, 23]. An arthroscopic study has shown that cyclops lesions develop from fibroproliferative tissue originating from remnants of the ACL stump [12]. A recent meta-analysis has shown that the main complications of the remnant-preserving technique were cyclops lesion and a loss of extension-ROM [29]. Figure 3 shows a cyclops lesion after ACL reconstruction and histological representation of typical dense connective tissue in all cyclops lesions, resemble to tendinous tissue. Several previous studies reported a high rate of cyclops lesions or roof impingement with extension loss after remnant-preserving ACLR [4, 8, 23]. On the other hand, other authors found no increased rate of Cyclops lesions or roof impingement in remnant-preserving ACLR [17]. It seems likely that the specific technique of remnant augmentation could be an influencing factor on the rate of cyclops lesions. Intraoperatively the remnant can be debrided with a shaver from inside the tibial tunnel to reduce the volume of the remnant. We hypothesize that the rate of cyclops lesions after remnant-preserving ACLR using this technique is not higher than that of conventional ACLR.

Therefore, it was aim of the present study to evaluate if there is a higher risk of motion complications and cyclops lesions in patients after ACLR using the remnant-sparing technique.

## Materials and methods

### Study design

Patients with ACL rupture who agreed to participate in the study were recruited to the study by one of the authors from 2015 to 2016. Patients were consecutive enrolled as soon as they consented.

The following inclusion and exclusion criteria were used to identify eligible patients.

Inclusion criteria:

- ACL reconstruction with quadriceps tendon or hamstring tendon graft
- primary cruciate ligament reconstruction

Exclusion criteria:

- Additional interventions such as open peripheral ligament reconstructions (ALL-plastic, remodeling osteotomies, cartilage transplants, meniscus suture)
- Age < 18 years and > 50 years

The patients decided on the basis of sporting claim and after consultation about the graft (semitendinosus or quadriceps tendon).

In particular, patients with meniscus suture were excluded as it is known that meniscus suture poses an increased risk to the development of arthrofibrosis [7]. Patients were included until the previously calculated group size was reached (*n* = 60). The study’s design was assessed by the local ethics committee (ethic vote nr.: EA1/004/15).

### Treatment groups

Allocation to the various treatment groups was based on the arthroscopic findings. Patients with sufficient residual tissue (Crain type 1–3) were assigned into group 1 “remnant augmentation” [11]. Patients with no identifiable ligament tissue remaining were assigned to group 2 “no remnant”. The time for surgery after ACL rupture is comparable in both groups and averages 5 weeks after the ACL rupture.

### Sample size calculation

The primary outcome parameter is the arthroscopic arthrolysis or resection of a cyclops syndrome. In the literature, the rate of arthroscopic arthrolysis due to ACL reconstruction is given at about 2% [27]. In order to achieve a statistically significant result with a 5% risk of a type 1 error, a minimum sample size of 60 patients per group should be expected at a performance of 80%.

### Surgical technique of ACL reconstruction

In all patients, first an arthroscopic examination of the knee was performed to assess the ACL remnant according to the Crain classification and for the presence of meniscal injuries. As described above, patients with meniscus refixations were excluded [7].

Except for the preservation of the remnants of the ligament, the same surgical technique was used in both groups. This anatomic technique for ACL reconstruction with medial portal drilling has been described in detail previously [25]. In both groups, dilators were used for tunnel preparation after initial drilling with a 6 mm drill bit. Graft fixation was performed with an extracortical button at the femoral side (Flipp tack, Karl Storz, Tuttlingen, Germany) and with an interference screw and an extracortical button at the tibial side (Megafix interference screw and endo tack, Karl Storz, Tuttlingen, Germany).

In the group with the remnant augmentation, as much old cruciate ligament tissue as possible was preserved. Only aberrant ligament remnants, which turned in anteriorly and impressed like a cyclops nodule, were resected (Fig. 1). The new ligament is placed over the tunnel layer exactly in the tibial cruciate ligament stump so that the old ligament encloses the new one. At the femoral side, the insertion was debrided only as much that the important landmarks for tunnel drilling became visible [26, 31].

After tunnel preparation at the tibial side, a synovial shaver was guided through the tibial tunnel and the stump was debrided from the inside to reduce the volume of the remnant (Fig. 1). After finishing a remnant augmentation, the tendon graft is ideally almost completely covered with old ligament tissue (Fig. 2). There were 3 surgeons who were all instructed and they operated both groups. The operating surgeons were already familiar with the surgery procedure before the study and were trained again before the start of the study. The study was conducted at only one study center.

### Rehabilitation

All patients were treated with a phase adapted and criteria-based rehabilitation scheme. Physiotherapy with lymphatic drainage began on the 1st postoperative day. Partial weight bearing with 10 kg was recommended for the first two weeks after surgery. After two weeks, full weight bearing was allowed. Range of motion was not restricted. Aim after 6 weeks was full extension and 120° of flexion.

### Outcome measures

Primary outcome measure was the rate of re-arthroscopy with cyclops resection after ACL reconstruction due to a postoperative extension deficit. The indication for re-arthroscopy was given when there was a persistent extension deficit of more than 10° after more than 4 months and if physiotherapeutic treatment was unsuccessful. The follow-up time was one year after surgery, since most arthrolyses or cyclops resections have to be performed during this time due to movement complications [7]. In all patients with an arthroscopic cyclops resection, the position of the tibial and femoral bone tunnel was checked on lateral X-rays according to the quadrant method stated by Bernard and Hertel for the femur and according statements of Petersen and Zantop at the tibia [6, 21, 26]. According to the quadrant method, the center of the femoral tunnel should be located at between 20 and 30% of the distance t measured from the most posterior contour of the lateral femoral condyle and between 25 and 35% of the height h measured from Blumensaat’s line. At the tibia, the center of the bone tunnel bundle should be located between 35 and 45% of the sagittal tibia diameter [21].

Secondary outcome measures were subjective complaints measured with the Knee Osteoarthritis Outcome Score (KOOS), and patient satisfaction (5-point Likert scale) and return to sports rate.

### Statistical analysis

The odds ratio between the groups was calculated to determine the increased risk of arthrolysis in the respective group and to determine the probability of an unsatisfactory outcome in the two groups.

Normal distributions of the primary endpoints (KOOS and NRS score) were tested with the Kolmogorov–Smirnov test. Significant intergroup differences were tested with an independent *t* test. If data were not normally distributed, the nonparametric Mann–Whitney U test was used.

All calculations were made using SPSS software (ver. 20.0, SPSS Inc., Chicago, IL).

## Results

### Patients

**Fig. 4:**
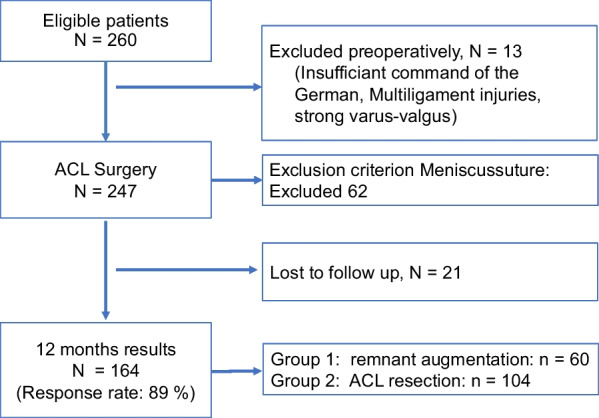
Patient selection and loss to follow-up

Figure 4 shows a flowchart of patient selection and loss to follow-up. One hundred and sixty-four patients met the inclusion criteria and could be included. 60 Patients received the “remnant augmentation” procedure (Table 1).

**Table 1:** Patient distribution

	Numbers	Gender		Age	Graft		Partial meniscus resection
		Female	Male		Hamstring	Quadriceps	
Remnant augmentation	60	29	31	34.8 ± 12.1	41	19	16
ACL resection	104	54	50	34.9 ± 12.3	90	14	28

### Primary outcome measure

Of the total 164 patients were included, and re-arthroscopy with cyclops resection was performed in 4 patients due to clinically relevant extension deficit (2.4%). In all these patients, a cyclops nodule was observed and resected. A graft rupture was not seen during the observation period.

One re-arthroscopy was performed in group one (remnant augmentation) and 3 in group two (remnant resection). Of the 4 re-arthroscopies, one patient had received a quadriceps tendon as a graft. This was in the group “ACL resection”. Table 2 shows an odds ratio of 0.6, which means ACL reconstruction with resection of the old cruciate ligament stump has a slightly higher risk of re-arthroscopy.

**Table 2:** Odds ratio for arthrolysis for remnant augmentation (OR 0.6)

	Arthrolysis	No arthrolysis	
ACL remnant	1	59	60
ACL resection	3	101	104
	4	160	**0.6**

Bold indicate not possible to calculate a p-value by odds ratio

The histological examination showed that dense connective tissue with a resemblance to tendinous tissue was detectable in all cyclops lesions (Fig. 2). Two nodules contained also chondroid tissue.

In all revision cases, the center of the tibial and femoral bone tunnel was located within the landmarks stated by Bernard and Hertel [6] and Petersen and Zantop [26].

### Secondary outcome measures

**Fig. 5:**
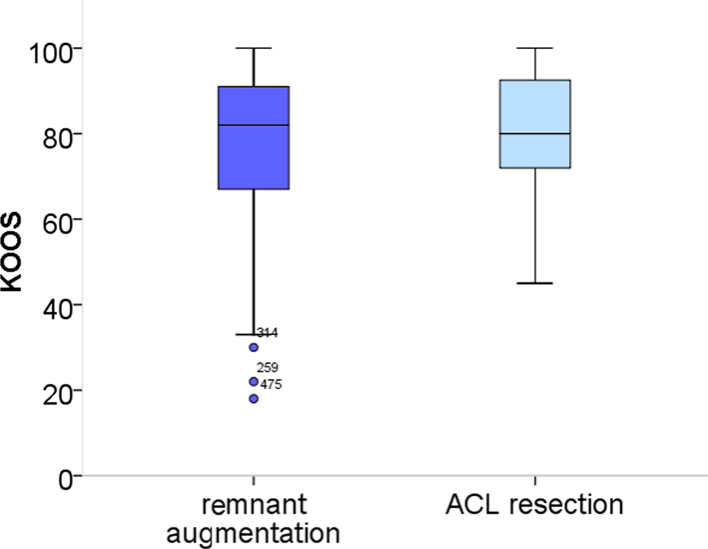
KOOS score in the remnant-preserving and non-remnant-preserving group. The difference was statistically not significant (*P* = 0.19)

**Fig. 6:**
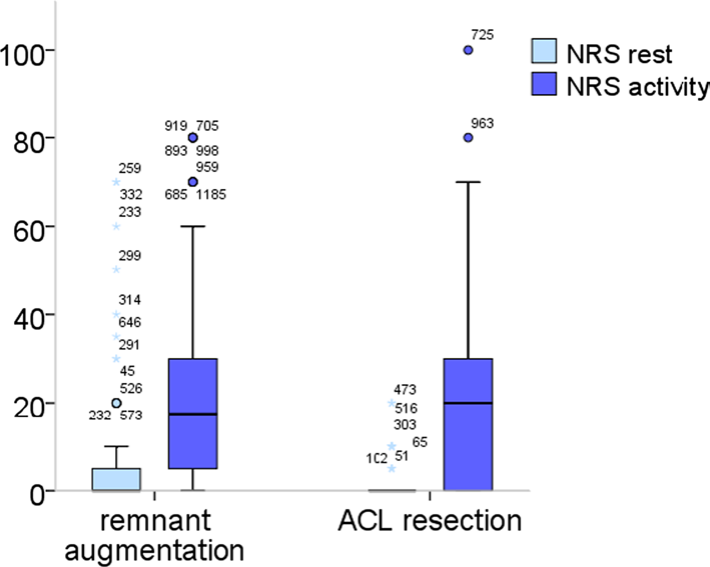
Pain (NRS) in the remnant-preserving and non-remnant-preserving group. The difference was statistically not significant (*p* = 0.51 rest, *p* = 0.41 activity)

**Fig. 7:**
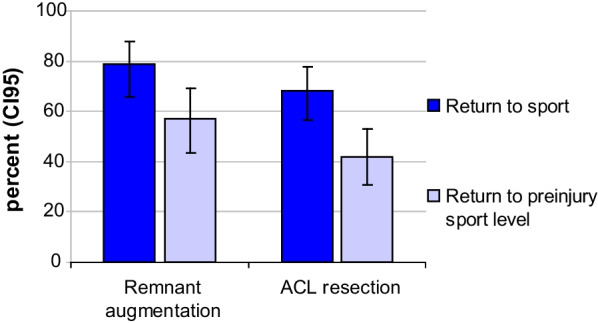
Return to sport and return to preinjury sport level 12 months after surgery, higher values in the remnant group

There was no significant difference in the secondary outcome criteria such as the KOOS (Fig. 5), pain (Fig. 6) and patient satisfaction (Table 3). The return to sport rate at one year after ACL reconstruction, however, was significantly higher in the remnant-preserving group (79% vs. 68%, Fig 7). In the “remnant augmentation” group, more athletes have already reached their previous sporting level (57% vs. 42%).

**Table 3:** Patient satisfaction (Likert scale), slightly higher satisfaction in the remnant augmentation group, but not significant (*p* = 0.373)

Satisfaction (in percent)	Very satisfied	Satisfied	Mediocrely satisfied	Unsatisfied	Very unsatisfied
Remnant augmentation	45	35	16	4	0
ACL resection	35	47	12	4	2

## Discussion

The results of the present study confirm the initial hypothesis. The rate of cyclops lesions after remnant-preserving ACL reconstruction was not any higher than after conventional ACL reconstruction. The odds ratio of 0.6 tends to show that ACL reconstruction with residual resection has a slightly higher risk of a cyclops lesion in the postoperative course. It could be that the old ACL stump has a protective effect on the graft.

It has been shown that the pathogenesis of cyclops lesions after ACL reconstruction is multifactorial [13, 28]. Typical causes for cyclops lesions can be debris raised during drilling of the tibial tunnel or partially torn fibers of the ACL graft on the intercondylar notch [13, 28]. Interestingly, in all cases, the histopathological examination revealed that the cyclops lesion was due to a torn bundle of the tendon graft. The ligamentous sleeve apparently prevents individual graft bundles from shearing off during remodeling and rehabilitation. But debris from the tibial tunnel could also be held back by the tibial ACL remnant tissue.

In addition, studies have shown that ACL remnant augmentation improves graft remodeling [18]. Animal studies could demonstrate that residual ACL tissue has the potential to accelerate graft remodeling [22]. Cells of human ACL remnants have the ability to synthesize collagen, and the synovial layer provides vascular support [20]. Kirizuki et al. [16] described that ACL remnants contain vascular stem cells that may contribute to ACL regeneration and repair. It has been shown by MRI that after 6 months already, the graft had the same signal intensity as the surrounding remnant material [23]. All of these findings indicate that preserving the remnant in ACL reconstruction improves tendon graft remodeling.

Graft revascularization and remodeling are known to be important for the biomechanical properties of the tendon graft. A faster graft remodeling could therefore also contribute to fewer bundle ruptures. This hypothesis is supported by a clinical study by Takazawa which has shown that preservation of the remnant decreased the likelihood of graft rupture [27].

The lower rate of graft rupture after remnant-preserving ACL reconstruction can also be attributed to improved proprioception. In the study by Ma et al., postoperative proprioception was significantly better after remnant-preserving ACL reconstruction than after conventional remnant-sacrificing ACL reconstruction [30]. It is speculated that preserving proprioceptive elements in the old stump leads to improved postoperative neuromuscular capabilities [18]. Due to the proprioception received, neuromuscular control improves and thus the risk of non-contact trauma could be reduced.

The difference to previous studies, which showed a higher type of cyclops lesions, may be due to the fact that in the present study only symptomatic cyclops lesions were counted, which required arthroscopic revision. In the study by Ahn et al., asymptomatic cyclops lesions were scored [4]. Another reason may be the surgical technique. A so-called remnant-sparing technique was used in the present study. In this procedure, an excessive increase in volume in the area of the notch is avoided.

In the present study, there was no difference in any other secondary outcome measure such as KOOS and pain and only a slight difference in the return to sports rate. This could be due to the short follow-up period of one year or due to the specific scores used in this study. However, the KOOS values determined were comparable to those from a recently published study on one-year results from the Swedish Cruciate Ligament Register [14]. A recent systematic review showed that remnant-preserving ACL reconstruction provided a superior outcome of postoperative KT 1000 and Lysholm score, whereas no difference could be found for “International Knee Documentation Committee” (IKDC) subjective score (International Knee Documentation Committee), IKDC grades or pivot shift tests [29].

Considering improved remodeling, fewer cyclops lesions, slightly better “patient-reported outcome measures” (PROMs), lower rerupture rate and higher stability, we believe that remnant-preserving ACL reconstruction should continue to be followed in the future. However, it must be noted that this is a very demanding surgical technique, since particularly the femoral insertion is not easily visible. Therefore, this technique is more suitable for experienced surgeons. Nevertheless, a recent study using three-dimensional CT evaluation has shown that despite the presence of a large remnant, an anatomic tunnel placement is possible when a remnant-preserving surgical technique is used [9]. In the present study, in all revision cases, the center of the tibial and femoral bone tunnel was located within the landmarks stated by Bernard and Hertel [6] and Petersen and Zantop [26] so that a non-anatomical tunnel position as cause of the movement deficit could be excluded.

The present study has some limitations. One limitation is that patients were not randomized to the different treatment groups. The lack of randomization could have led to a bias in patient selection. Patients were allocated to the groups based on the integrity of the remaining cruciate ligament. The preoperative stability in the group without receiving the remnant may have been lower, since it is known that the remnant contributes to the stability of the knee joint. However, preoperative stability is not a factor that affects the primary endpoint of the present study (rate of cyclops lesions and anterior impingement problems). The value of randomized trials may at times be overstated. A Cochrane review study compared the reliability of observational studies with that of randomized controlled trials [5]. In this systematic review, the differences were not as significant as previously believed [5]. These authors recommended that more attention should be paid to prevent judging of research based only on study types [5]. One pitfall of randomized controlled trials, for example, is selection bias because many patients refuse to be included in a controlled trial [24]. Therefore, the results of these trials cannot be transferred to a real-world scenario, but in spite of all RCTs still are considered as the gold standard. Another limitation could be the short follow-up time of only one year. Many experts require a minimum follow-up of two years for knee studies. In this case, however, a longer follow-up would not have had a major impact on the primary outcome criterion, since complications such as motion complications after cruciate ligament plastic usually manifest in the first postoperative year.

## Conclusions

In conclusion, patients who have undergone the sparing “remnant augmentation” ACLR have no increased risk of cyclops lesion formation or extension deficit in the first year after surgery. An improvement of the proprioceptive abilities by remnant augmentation ACLR should be investigated in further studies.

## Data Availability

The data can be called up upon written request.

## Abbreviations

ACL: Anterior cruciate ligament
ACLR: Anterior cruciate ligament reconstruction
ALL: Anterior lateral ligament
IKDC: International Knee Documentation Committee
KOOS: Knee Osteoarthritis Outcome Score
MRI: Magnetic resonance imaging
NRS: Numeric rating scale
PROMs: Patient-reported outcome measures
